# Oral potentially malignant disorders: Is malignant transformation predictable and preventable?

**DOI:** 10.4317/medoral.20205

**Published:** 2014-06-06

**Authors:** Isaäc van der Waal

**Affiliations:** 1 Department of Oral and Maxillofacial Surgery/Pathology and Academic Centre for Dentistry (ACTA), Amsterdam

## Abstract

Leukoplakia is the most common potentially malignant disorder of the oral mucosa. The prevalence is approximately 1% while the annual malignant transformation ranges from 2% to 3%. At present, there are no reliable clinicopathological or molecular predicting factors of malignant transformation that can be used in an individual patient and such event can not truly be prevented. Furthermore, follow-up programs are of questionable value in this respect. Cessation of smoking habits may result in regression or even disappearance of the leukoplakia and will diminish the risk of cancer development either at the site of the leukoplakia or elsewhere in the mouth or the upper aerodigestive tract. The debate on the allegedly potentially malignant character of oral lichen planus is going on already for several decades. At present, there is a tendency to accept its potentially malignant behaviour, the annual malignant transformation rate amounting less than 0.5%. As in leukoplakia, there are no reliable predicting factors of malignant transformation that can be used in an individual patient and such event can not truly be prevented either. Follow-up visits, e.g twice a year, may be of some value. It is probably beyond the scope of most dentists to manage patients with these lesions in their own office. Timely referral to a specialist seems most appropriate, indeed.

** Key words:**Oral potentially malignant disorders, oral leukoplakia, oral lichen planus.

## Introduction

At present, preference is given in the literature to the use of the adjective “potentially malignant” rather than to premalignant or precancerous. Furthermore, in case of leukoplakia one favors to use “disorder” instead of lesion as was done in the past, recognizing the fact that malignant transformation does not always take place in the leukoplakic area, but also elsewhere in the mouth or even elsewhere in the upper aerodigestive tract.

The following disorders are regarded as being potentially malignant: 1) leukoplakia/erythroplakia, 2) submucous fibrosis, 3) palatal lesions in reverse smokers, and, although still somewhat questionable 4) lichen planus, and 5) discoid lupus erythematosus. In addition, in patients suffering from rare, inherited syndromes such as xeroderma pigmentosum and Fanconi’s anemia, there is an increased incidence of oral cancer. This is also the case in immunodefiency, e.g. due to the prolonged use of immunosuppressive drugs or due to an underlying HIV-infection. Oral cancer has also been reported in patients suffering from chronic Graft Versus Host Disease after stem cell transplantation.

In this paper, the emphasis will be on leukoplakia/erythroplakia and lichen planus. In several recent reports in the literature the subject of leukoplakia and lichen planus has been combined under the heading of potentially malignant disorders, not clearly making a distinction between these two entities. In the present report, these two entities will be delt with separately.

## 1. Leukoplakia and erythroplakia

In the past decades little progress has been made in defining oral leukoplakia. In 1978, the World Health Organisation defined leukoplakia as “a white patch or plaque that cannot be characterized clinically or pathologically as any other disease” (1). In that communication it was noted, that the term leukoplakia was unrelated to the absence or presence of epithelial dysplasia. In 2005, the World Health Organisation defined leukoplakia as “a white plaque of questionable risk having excluded (other) known diseases or disorders that carry no increased risk for cancer” (2). Both the 1978 and the 2005 definition are worded in a somewhat negative way. Besides, when accepting the view that lichen planus is a potentially malignant disorder (to be discussed later), then this disease actually falls within the 2005 WHO definition of leukoplakia.

The prevalence of leukoplakia for all ages is approximately one per cent, with an increasing prevalence in adults. The male-female ratio varies in different parts of the world. Smoking is the most common etiologic factor. Nevertheless, leukoplakia may occur in non-smokers as well. Cessation of smoking habits may result in regression or even disappearance of the leukoplakia in a matter of a few months.

When the incidence of oral cancer is set at 5 per 100.000 population per year, then an annual risk of malignant transformation in oral leukoplakia patients of 2% is a four hundred times increased risk. Apparently, this figure qualifies for a “significantly” increased risk.

Prevalence figures of erythroplakia are only available from studies in South- and South East Asia and are as low as 0.02% (3). The annual malignant transformation rate is actually unknown but is much higher than in leukoplakia. Because of its rarity, this entity will not be discussed here any further.

1.1- Risk factors of malignant transformation in leukoplakia 

In general, it seems well accepted that the annual malignant transformation rate of leukoplakia amounts 2%-3% for all clinical subtypes together, including the ill-defined and much debated entity of proliferative verrucous leukoplakia.

There are numerous reported parameters that allegedly predict future malignant transformation of oral leukoplakia. These parameters include previously diagnosed cancer in the head and neck region, older age, female gender, absence of smoking habits, duration of the leukoplakia, clinical subtype (homogeneous versus non-homogeneous), large seize, and oral subsite such as borders of the tongue and floor of the mouth (4). The use of toluidine blue staining may help to identify high-risk leukoplakias with poor outcome (5). Other predicting factors include the presence of C. albicans, the presence and severity of epithelial dysplasia, and, in addition, numerous molecular markers, such as aberrant expression of p16INK4a and Ki-67, (6) chromosome instability, (7) and loss of heterozygosity at 9p and mutated TP53 (8).

Some of the predicting factors mentioned above carry a certain degree of subjectivity. For instance, it may difficult to objectively define the clinical homogeneous subtype. Some clinicians use this adjective only in case of thin , smooth and homogeneously white lesions, while others may apply this adjective also in homogeneously white and homogeneously verrucous lesions. The histopathological assesment of the presence of epithelial dysplasia and the degree of dysplasia is another source of substantial subjectivity.

**Figure 1:**
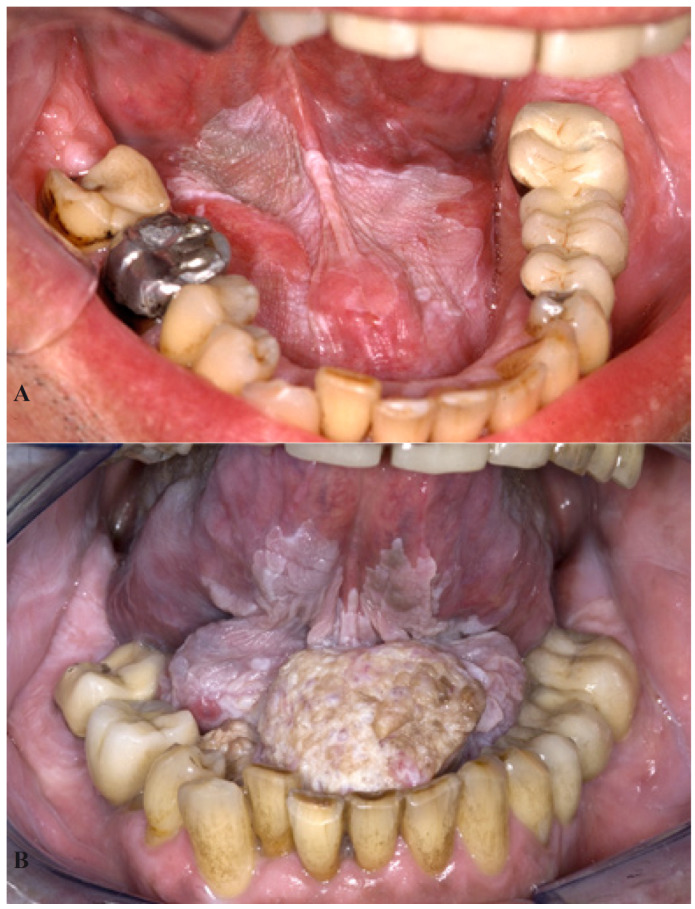
Homogeneous leukoplakia in a 57-year-old man A). A biopsy showed hyperkeratosis without epithelial dysplasia. The patient was unable to stop smoking and refused any type of treatment. He was lost to follow-up and showed up 12 years later with a large squamous cell carcinoma B).

At present, there is no single marker or set of markers that reliably can be used to predict malignant transformation of oral leukoplakia in an individual patient. Statistically, when applied to a large group of patients, non-homogeneous clinical subtype, large seize, presence of epithelial dysplasia and location on the tongue or the floor of the mouth (at least in the Western world; in India the buccal mucosa is the most common oral subsite at risk) seem to be the most relevant predictors of malignant transformation (Fig. 1).

1.2- Treatment and follow-up

The main treatment modalities for oral leukoplakia can be divided in surgical treatment, including lasers, and non-surgical treatment.

While in oncologic surgery a margin of 1 cm beyond the visible or palpable extent of the oral cancer is a widely accepted guideline, no such guideline is available for the treatment of leukoplakia. Besides, in many leukoplakias there is no sharp delineation, thereby hindering to extent the excision or laser evaporation well into normal appearing mucosa.

**Figure 2:**
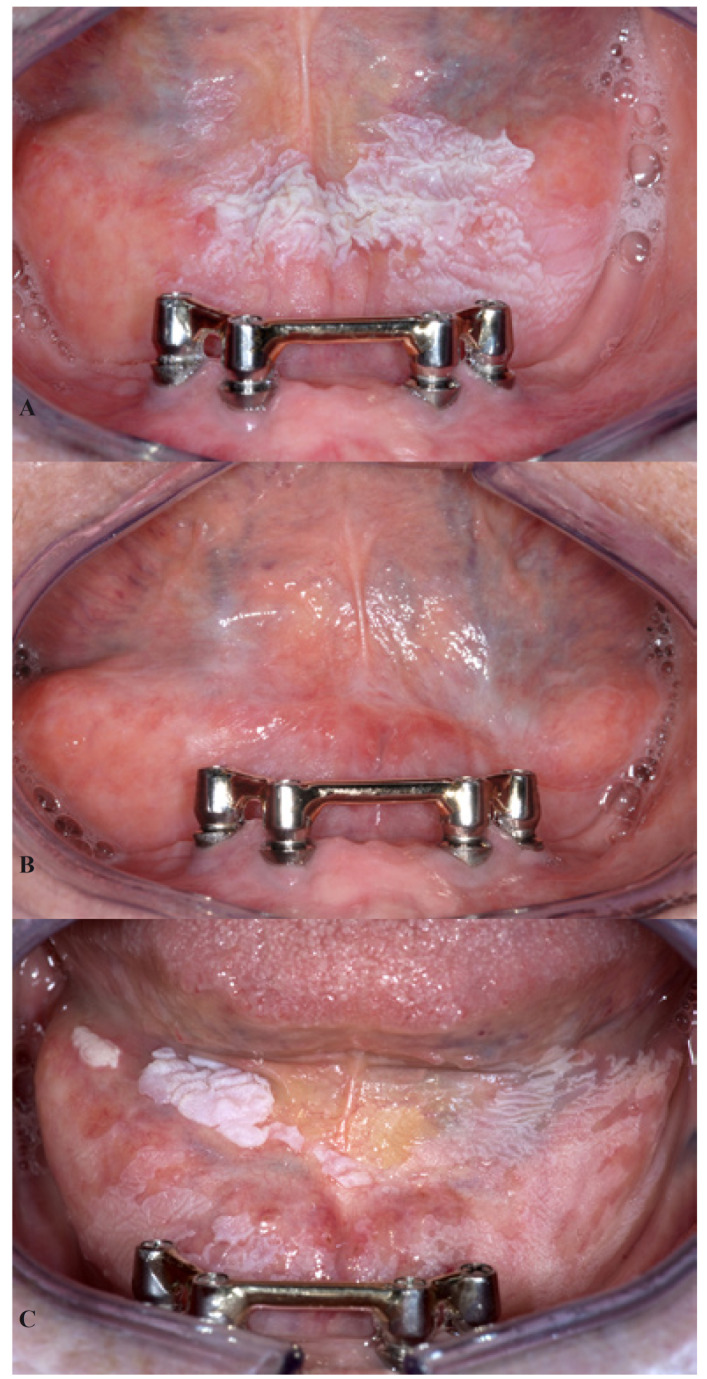
A 61-year-old woman with homogeneous leukoplakia in the floor of the mouth A). A biopsy showed hyperkeratosis without epithelial dysplasia. Treatment consisted of CO2 laser evaporation, resulting in an apparently good result after 6 months B). Six years later the leukoplakia had recurred C).

**Figure 3:**
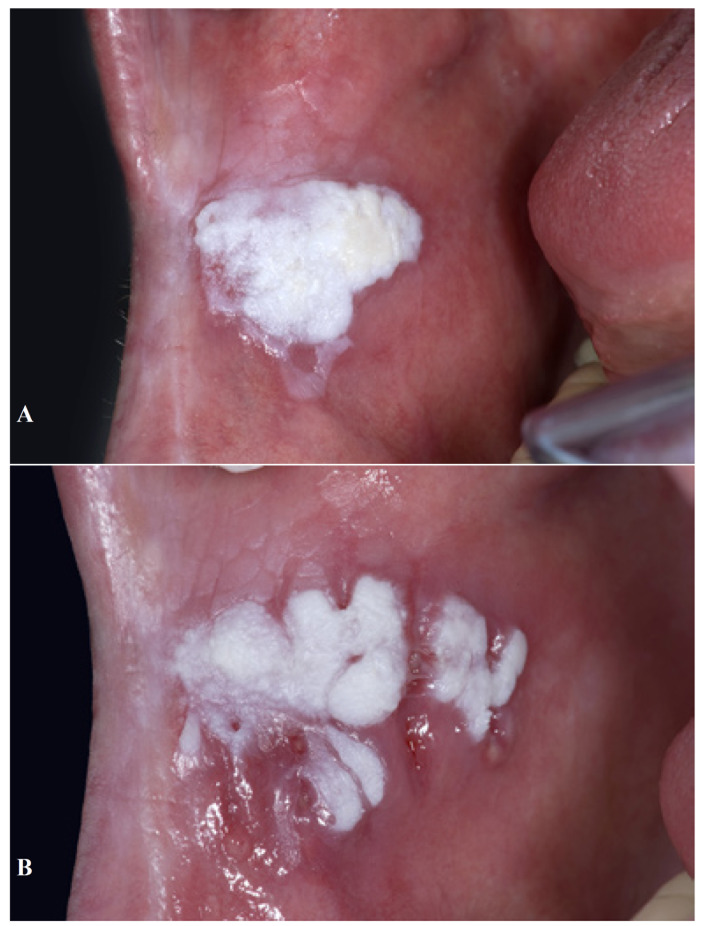
A 63-year-old man with verrucous leukoplakia of the buccal mucosa A). The leukoplakia recurred within three weeks after surgical removal B).

Local recurrences after surgical treatment, including lasers, are not uncommon (Figs. 2,3), the annual recurrence rate being approximately 5%-10% (9,10). In spite of an occasional retrospective study suggesting the opposite, (11) surgical treatment of leukoplakia does not seem to reduce the risk of future development of oral cancer either at the site of the leukoplakia or at another site in the oral cavity or the upper aerodigestive tract. Such observation has been made in several recent studies, e.g. by Arduino *et al*. (12) and Brouns *et al*. (13) but also in studies performed in the sixties of the past century. (14) The same applies to the result of non-surgical treatment (15,16).

There is no evidence that lifelong follow-up programs in treated or untreated patients with leukoplakia are effective in preventing the development of oral cancer. Most likely, follow-programs will not result in improved survival in case of cancer development either.

Cessation of smoking habits considerably reduces the risk of developing cancer after surgical treatment of oral potentially malignant lesions (17).

1.3- Patients’ management

Although removal of leukoplakia most likely will not eliminate or even reduce the risk of future development of oral cancer, most patients probably will prefer to have the leukoplakiaa removed, if feasible, when balanced against the morbidity of the treatment. In case of oral cancer development in a solitary, small (2cm-3cm) and well-circumscribed leukoplakia, the patient will, conceivably, regret the decision of non-treatment in such instance. Also the clinician will probably feel “guilty” of not having recommended treatment in such cases.

In case of non-treatment, most patients prefer to be followed-up, in spite of the questionable efficacy of such follow-up. Depending on various aspects, such as the extent of the leukoplakia and the presence and degree of epithelial dysplasia, intervals may vary from 3-6 months, lifelong. Changes in the clinical manifestation and, particularly, symptoms are ominous signs of malignant transformation.

It is probably beyond the scope of most dentists to manage patients with oral leukoplakia in their own office. Instead, timely referral to a specialist seems most appropriate.

## 2. Oral lichen planus

The prevalence of oral lichen planus is in general accepted to be approximately 1 per cent. This chronic disorder mainly affects middle-aged people. The etiopathogenesis is still poorly understood. There is no effective treatment and there are no preventive measures either.

The debate on the allegedly potentially malignant character of oral lichen planus is going on already for several decades (18-23). In a seven-year follow-up study of 327 patients the annual maligant transformation rate amounted less than 0.5% (24). When the incidence of oral cancer is set at 5 per 100.000 per year, then an annual risk of malignant transformation in oral lichen planus patients of 0.5% is a hundred times increased risk. Is this sufficient for qualification as a “significantly increased risk”? The cancer may develop anywhere in the oral cavity, not necessarily at the site of the lichen planus.

An important obstacle in the discussion on the possible potentially malignant character of oral lichen planus is caused by the lack of clear clinical and histopathologic diagnostic criteria of oral lichen planus, resulting in a poor clinicopathologic correlation in the diagnosis (25). In daily practice it is, indeed, sometimes impossible to reliably make a distinction between the various clinical manifestations of lichen planus and those of leukoplakia, both clinically and histopathologically. Another area of confusion is the recognition of so-called lichenoid lesions, e.g. amalgam related lesions.

2.1- Risk factors of malignant transformation in lichen planus

There are no known clinical or histopathologic features in oral lichen planus that predict possible maligant transfromation. In general, the presence of epithelial dysplasia is not accepted within the diagnostic spectrum of oral lichen planus. In this respect, the term “lichenoid dysplasia” is a rather confusing one (26). There are only a few studies on molecular markers that might be of predicting value with regard to malignant transformation in oral lichen planus (27-29). At present, no such markers have shown to be of strong predictive power.

2.2- Follow-up programs

The efficacy of continous, lifelong follow-up of patients with oral lichen planus is questionable, (30) although structured follow-up visits have been suggested to be beneficial in some studies (31).

2.3- Patients’ management

In daily practice it may be difficult to manage patients with oral lichen planus with regard to the debatable issue of premalignancy, even when the annual malignant transformation is set at a percentage of less than 0.5 per cent. There is no effective treatment for lichen planus, there are no known predictive factors of malignant transformation, it is not possible to prevent future cancer development and the efficacy of follow-up is at least questionable. The general advice in these circumstances is to perform an oral examination at least once a year, preferably twice a year. These examinations could be performed by dentists in their own office without the need for routine referral to a specialist.

## Conclusion

At present, there are no reliable predicting factors of malignant transformation that can be used in an individual patient with oral leukoplakia and such event can not truly be prevented. Furthermore, follow-up programs are of questionable value.

At present, there is a tendency to accept that oral lichen planus is a potentially malignant disorder, the annual malignant transformation rate amounting less than 0.5%. As in leukoplakia, there are no reliable predicting factors of malignant transformation that can be used in an individual patient and such event can not truly be prevented either. Follow-up visits, e.g twice a year, may be of some value.

It is a challenge for the dentists to convey the views on oral potentially malignant disorders to their patients. Some patients will be able to draw their own conclusions and to properly balance the aspects of the morbity of treatment and the questionable benefit of such treatment. Others may not be able to oversee the various aspects of potentially malignant disorders and the lack of prediction and prevention of malignant transformation. In case of leukoplakias that are localized and well-circumscribed removal seems advisable in such instances. In any event, follow-up is recommended in spite of its questionable value.

In case of lichen planus follow-up at 6-12 months, lifelong, seems a reasonable advise.

Particularly in case of oral leukoplakia most dentists will probably feel the need to refer the patient to a specialist for diagnostic reasons and, even more so, for determining the management policy.
